# Radiological and intraoperative findings of a rare case of vestibular paroxysmia

**DOI:** 10.1007/s10072-024-07695-2

**Published:** 2024-07-18

**Authors:** Nicola Montano, Eleonora Ioannoni, Alessandro Izzo

**Affiliations:** 1 Department of Neuroscience, Neurosurgery Section, Università Cattolica del Sacro Cuore, Largo Agostino Gemelli, 8, Rome, 00168 Italy; 2 Neurosurgical Intensive Care Unit, Fondazione Policlinico Universitario Agostino Gemelli IRCCS, Università Cattolica del Sacro Cuore, Largo Agostino Gemelli, 8, Rome, 00168 Italy

**Keywords:** Vestibular paroxysmia, Neurovascular conflict, Microvascular decompression, Magnetic resonance imaging, Cerebello-pontine angle, Diagnosis, Outcome

## Abstract

Vestibular paroxysmia (VP) is a rare condition. The pathogenesis is linked to a neurovascular conflict (NVC) between an abnormal arterial loop and the VII/VIII cranial nerve complex in the cerebello-pontine angle. Due to its rarity, intraoperative findings are only anecdotally reported. Here we reported on a case of VP, showing the radiological images and the intraoperative surgical video of microvascular decompression (MVD). Further we discussed our findings considering the pertinent literature. We think that in case of VP the concordance between the side of tinnitus/hypoacusia and the side of NVC on magnetic resonance imaging should be always looked for before considering MVD as a therapeutic option.

**Supplementary Information:**

The online version contains supplementary material available at 10.1007/s10072-024-07695-2.

Vestibular paroxysmia (VP) is a rare condition and its exact incidence is unknown [1]. Differently from other neurovascular conflict (NVC) syndromes such as trigeminal neuralgia and hemifacial spasm, there is no clear indication about the patients who might benefit from microvascular decompression (MVD). Furthermore, due to its rarity intraoperative findings are only anecdotally reported. The purpose of this paper is to report a paradigmatic case of VP showing the radiological features and an intraoperative video of the surgical technique. To the best of our knowledge, no previous intraoperative video of VP have been reported in a peer-reviewed scientific journal (we were able to find only one video published in a youtube personal channel at following link: https://www.youtube.com/watch?v=eD2-cWLkSdE).

A 53-year old female was admitted with three years history of paroxysmal recurrent vertigo. During the vertigo attacks, she complained of left tinnitus. She had had no response to different antivertigo drugs but partial response to carbamazepine (dosage of 600 mg/day at the beginning increased to 1000 mg/day in the last few months before the admission). On admission, she complained of at least 20 attacks per day with a poor quality of life. Magnetic resonance imaging (MRI) evidenced a possible left NVC, classified as type II conflict [2], as the artery entered but not extended more than 50% of the length of the internal acoustic meatus (Fig. 1). She underwent MVD of VIII cranial nerve under neurophysiological neuromonitoring [3]. During the operation, we found an abnormal arterial loop of the anterior-inferior cerebellar artery (AICA) crossing the VII/VIII cranial nerve complex that was dissected and pushed downward. A teflon pad was finally placed (Video, see Supplemental Material). In the post-operative, she developed a mild left hypoacusia with no other neurological deficit. At 1-year follow-up she is free from vertigo and from tinnitus with good general condition without any drug.

**Fig. 1:**
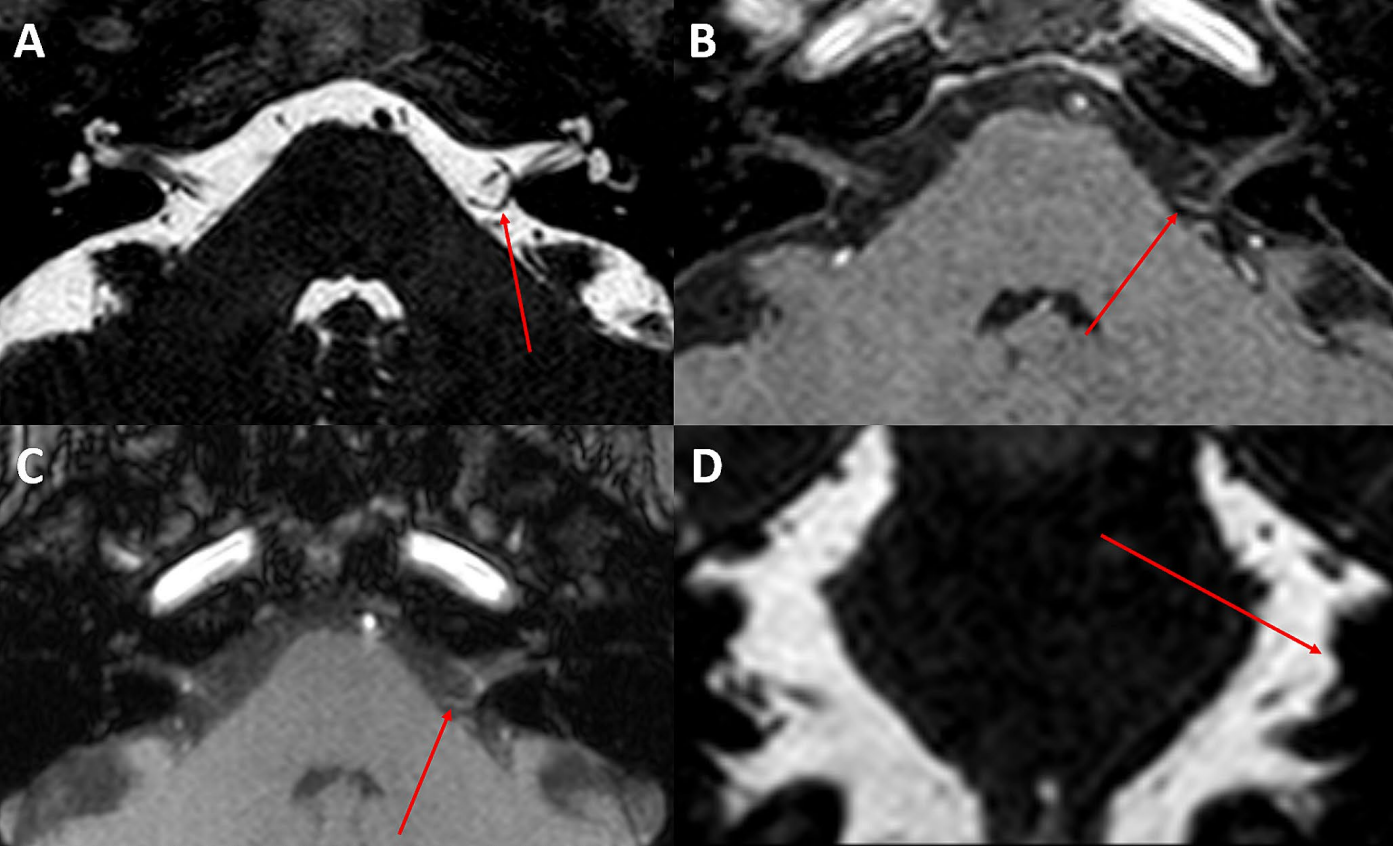
Brain MRI. (**A**) Axial T2 FIESTA, (**B**) T1-weighted post-gadolinium volumetric, (**C**) axial time-of-flight (TOF), (**D**) coronal T2 FIESTA sequences, showing a possible NVC between an abnormal loop of the AICA and the VIII cranial nerve (red arrow). In our case, there was a concordance between symptoms (mainly the left tinnitus) and the NVC side

VP is a difficult diagnosis. The Classification Committee of the Bárány Society identified the following criteria for the diagnosis of VP: (1) at least ten attacks of spontaneous spinning or non-spinning vertigo; (2) duration less than 1 min; (3) stereotyped phenomenology in a particular patient; (4) response to a treatment with carbamazepine/oxcarbazepine; (5) not better accounted for by another diagnosis [1]. Furthermore, before offering MVD other causes of vertigo should always be ruled out. Menière’s disease, paroxysmal brainstem attacks, superior oblique myokymia, vestibular migraine and benign paroxysmal positional vertigo should be considered in the differential diagnosis [1, 4]. It is important to note that in the reported diagnostic criteria there are no factors that can suggest the side of vascular compression. This is a problem because the suspect of NVC on one side on MRI is not enough to propose a MVD in these patients. In our case, the decision to operate was based on different factors. The patient complained of recurrent attacks of paroxysmal, non-positional and short lasting vertigo. The vertigo attacks were associated to the presence of a left tinnitus. The response (although temporary and partial) to antiepileptic drugs suggested a possible NVC etiology (like other NVC syndromes such as trigeminal neuralgia and hemifacial spasm). The concordance with the side of NVC and the side of tinnitus was another element that reinforced the possible causal relationship between the NVC and the vertigo attacks. The patient’s decision, due to long history and the failure of the conservative treatments, was another determinant factor leading to the surgical option.

In conclusion, like other NVC syndromes, we think that the MRI findings should always be analysed in the right clinical context. While it is easy to understand the affected side in trigeminal neuralgia and hemifacial spasm, in VP we always investigate the presence of a monolateral tinnitus or hypoacusia. The concordance between the side of tinnitus/hypoacusia and the side of NVC on MRI should be always looked for before considering MVD as a therapeutic option in these cases.

## Electronic supplementary material

Below is the link to the electronic supplementary material.

Supplementary Material 1
